# Surface shape alters perceived material softness

**DOI:** 10.1177/20416695241245021

**Published:** 2024-04-11

**Authors:** Hongbo Wang, Qingyu Sun, Shogo Okamoto

**Affiliations:** 12944 Tokyo Metropolitan University, Tokyo, Japan; 13143 University of Tokyo, Tokyo, Japan; 12944 Tokyo Metropolitan University, Tokyo, Japan

**Keywords:** hardness, texture, elasticity

## Abstract

When a human strokes the surface of an object with his/her finger, the surface shape influences the perceived softness of the object. This study introduced a curved surface softness illusion, which alters the perception of material softness. When a surface with curvature is felt by sliding a finger over it, it feels softer than a flat surface made of the same material. In contrast, a rugged surface is perceived as harder. This illusion indicates that, in addition to mechanical hardness, humans judge an object’s softness based on its surface shape.

Many researchers believe that pressing is the most common and effective motion for exploring the softness of an object. However, to investigate softness, humans employ stroking or rubbing motions as frequently as pressing motions (Arakawa et al., 2021; Cavdan et al., 2021). When a human strokes the surface of an object with his/her finger, the surface topology also influences the softness felt by the object. Thus far, the influence of surface shape on softness perception has not been demonstrated. The current study demonstrated the “curved surface softness illusion,” where objects with different surface features feel differently soft even if they are made of the same material.

We created four three-dimensionally printed plastic specimens, as shown in Figure 1 and Table 1. Two of these specimens were flat and smooth, but different in terms of material hardness. One was as hard as a plastic eraser (Young’s modulus: 2.46 MPa), and the other was as hard as a bouncing rubber ball (9.35 MPa). The other two specimens were composed of a resin with Youngs’ modulus of 9.35 MPa and exhibited sinusoidal surface displacements with distinct wavelengths ( λ in mm) and amplitudes ( A in mm). The values for one specimen were λ=10 mm and A=0.75 mm. The values for the other specimens were λ=50 mm and A=0.5 mm.

**Figure 1.**
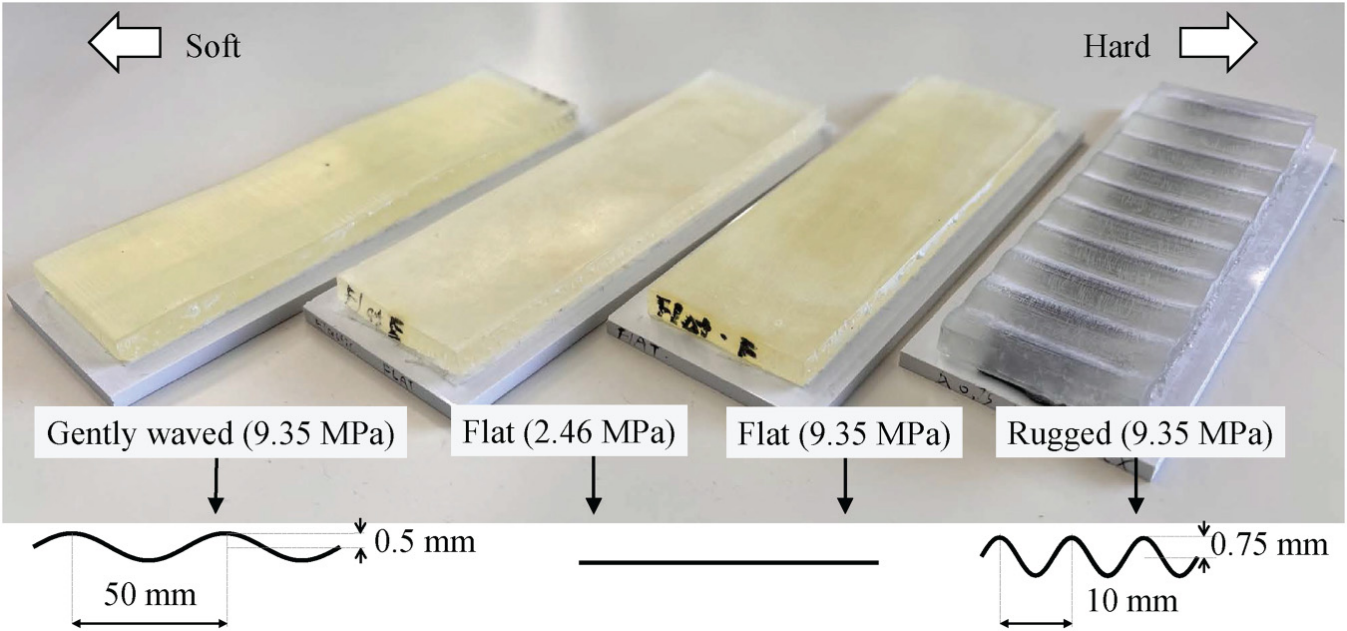
Samples arranged from softest to hardest (left to right). From left: gently waved (9.35 MPa), flat and soft (2.46 MPa), flat (9.35 MPa), and rugged (9.35 MPa).

**Table 1.** Stimuli parameters.

Softness	Specimen	Amplitude	Wavelength	Young’s
rank		A (mm)	λ (mm)	modulus (MPa)
1	Gently waved	0.5	50	9.35 (bouncing ball level)
1	Flat and soft	—	—	2.46 (eraser level)
3	Flat	—	—	9.35 (bouncing ball level)
4	Rugged	0.75	10	9.35 (bouncing ball level)

We asked 15 university students to rank the four specimens in the order of their perceived softness. Blindfolded participants stroked the randomly arranged specimens with their index fingers to probe their softness without pressing. No reference speed and contact force were provided. The specimens were lubricated with talcum powder to eliminate the differences in surface adhesion.

As shown in Figure 2, all the participants judged the flat specimen with Young’s modulus of 2.46 MPa to be softer than that of 9.35 MPa. The gently waved specimen felt softer than the flat specimen made of the same material (14 participants). Statistical significance was determined for each of these two pairs. Furthermore, the rugged surface felt harder than the flat specimen made of the same material (12 participants). Nine participants judged the gently waved surface to be softer than a flat surface made of the softest material. Hence, they largely agreed on the order of softness as follows: gently waved surface with Young’s modulus of 9.35 MPa, flat surface of 2.46 MPa, flat surface of 9.35 MPa, and rugged surface of 9.35 MPa.

**Figure 2.**
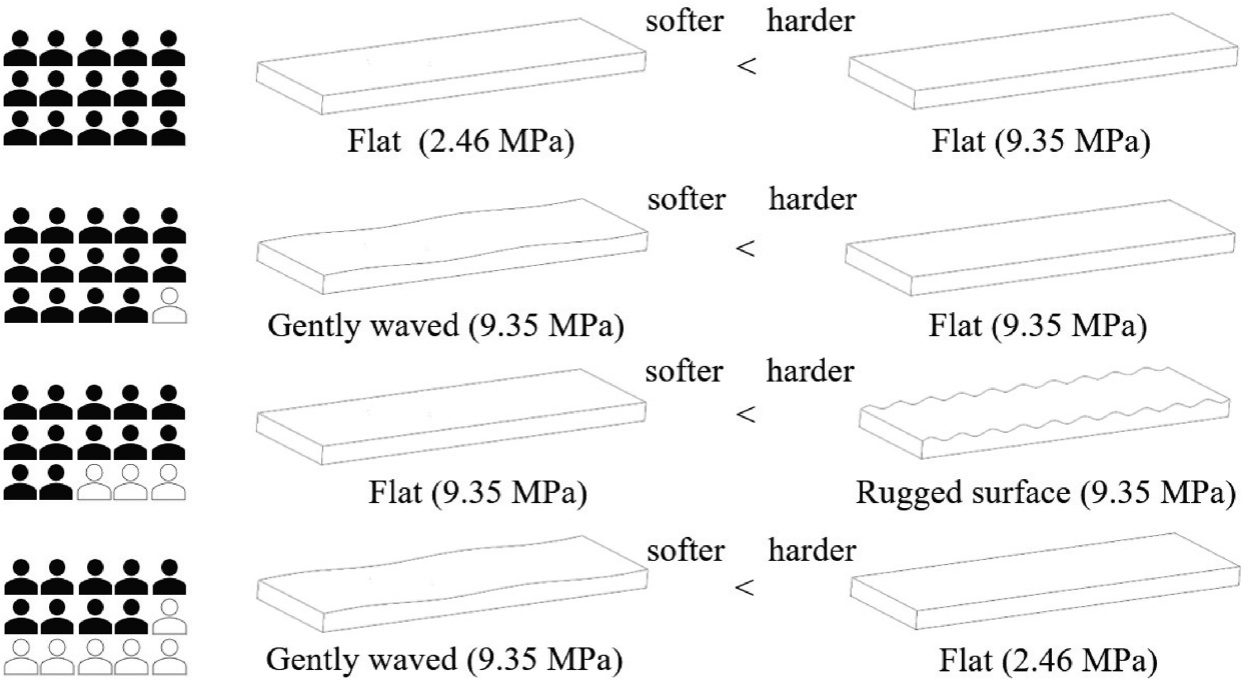
Summary of the trends in softness judgment. Filled human figures indicate the number of people in agreement with softness inequality.

The question here is what caused these results. There are several possible explanations for this.

- Contact area and softness judgment. The above effect may be considered similar to the finger-sized bump illusion (Inoue et al., 2022), where pressing a finger-sized concave surface with a fingertip causes it to feel softer and a finger-sized convex surface to feel harder than a flat surface. Researchers have explained that a surface with a larger contact area feels softer, whereas evoking a smaller contact area feels harder. When a fingertip glides over a sinusoidal displacement of λ=10 mm, which is smaller than the width of the fingertip, the entire area of the finger pad does not fit into the sinusoidal groove, resulting in a small real contact area. Hence, rugged surfaces can be felt harder. However, the gently waved specimen, which feels softer than the flat surface, comprises both concave and convex surfaces that are slightly larger than the fingertip. Therefore, the finger-sized bump illusion does not fully explain the softness experienced when stroking such surface profiles.
- Influence of slow changes in friction on softness. When a finger slides on a bumped surface, its gradient is associated with alterations in the tangential resistive force (Robles-De-La-Torre & Hayward, 2001). According to an experiment conducted by Ito et al. (2017), such a variation in force may evoke a sense of softness. However, their experiment does not explain why most participants judged rugged surfaces to be hard.
- Perceptual-linkage between roughness and softness. Hardness and roughness can be associated with each other through common sound symbols (Eitan & Rothschild, 2010). For example, high-pitched sounds evoke sensations of roughness and decreased softness. If these two distinct features share a similar multisensory process, it is conceivable that they can be conceptually mixed. It is important to note that the present experiment does not involve sounds, and the rugged surfaces used in our experiment suggest hardness without the involvement of sounds.

As mentioned above, the existing knowledge partly explains the effects of curved surfaces on softness judgment; however, the precise mechanisms underlying this experience remain unclear. Multiple principles may interact with or offset each other under specific conditions, leading to the complex mechanisms underlying this illusion. Further experimental validation is required to understand the effects of surface profiles on softness judgment.

This study found that the surface profile affected the perception of softness when stroked by a fingertip. Thus, the findings of this study shed light on human haptic perception and may also have implications in the field of product design.
